# Effect of dexmedetomidine administration on analgesic, respiration and inflammatory responses in patients undergoing percutaneous endoscopic lumbar discectomy: a prospective observational study

**DOI:** 10.1186/s12871-022-01691-9

**Published:** 2022-05-18

**Authors:** Xiaoli Zhang, Wenping Zhao, Cong Sun, Zhihua Huang, Lifang Zhan, Chunlin Xiao, Luying Lai, Reai Shan

**Affiliations:** 1 First Clinical Medical College, Gannan Medical University, Jiangxi, People’s Republic of China; 2 Department of Anesthesiology, Jiangxi Provincial People’s hospital, Jiangxi, People’s Republic of China; 3 Key Laboratory of Prevention and Treatment of Cardiovascular and Cerebrovascular Diseases of Ministry of Education, Gannan Medical University, Jiangxi, People’s Republic of China; 4 Department of Anesthesiology, First Affiliated Hospital of Gannan Medical University, Jiangxi, People’s Republic of China; 5 Department of Orthopaedics, First Affiliated Hospital of Gannan Medical University, Jiangxi, People’s Republic of China; 6 Department of Anesthesiology, Zhujiang Hospital, Southern Medical University, Guangzhou, People’s Republic of China; 7 Pain Institute, Jiangxi, People’s Republic of China

**Keywords:** Dexmedetomidine, Respiratory volume monitor (RVM), Percutaneous endoscopic lumbar discectomy (PELD), Inflammatory biomarkers, Oxidative stress biomarkers

## Abstract

**Background:**

Local anesthesia has been recommended for percutaneous endoscopic lumbar discectomy (PELD) in recent years; however, the efficacy, including oxidative stress, inflammatory reactions and ventilation effects, when intravenous dexmedetomidine (DEX) is administered during PELD has not been described.

**Methods:**

Sixty adult patients undergoing PELD were randomly allocated to either an intravenous DEX sedation group (Group A) or a normal saline group (Group B). Respiratory data, including minute ventilation (MV), tidal volume (TV), and respiratory rate (RR), were recorded using a respiratory volume monitor (RVM), and peripheral oxygen saturation (SpO_2_) was monitored by pulse oximetry. The visual analog score (VAS) was used to assess the level of pain. The serum levels of inflammatory biomarkers including interleukin-6 (IL-6) and tumor necrosis factor-α (TNF-α) were to assess inflammatory reactions. The serum levels of oxidative stress biomarkers including malondialdehyde (MDA) and glutathione peroxidase (GSH-PX) were also recorded to evaluate oxidative stress.

**Results:**

There were no significant differences in RR, MV, TV and SpO_2_ between the two groups at any time point (*P* > 0.05). Group B exhibited lower serum levels of GSH-PX (*P* < 0.0001) and higher serum levels of MDA (*p* < 0.0001) than Group A at the end of surgery. Twenty-four hours after surgery, Group B exhibited higher serum levels of IL-6 (*P* = 0.0033), TNF-α (*P* = 0.0002), and MDA (*P* < 0.0001) and lower serum levels of GSH-PX (*P* < 0.0001) than Group A. In addition, Group A exhibited lower VAS (*P* < 0.0001) than Group B during surgery.

**Conclusions:**

DEX administration using RVM not only provides analgesia without ventilatory depression but also alleviates oxidative stress and inflammatory reactions in patients undergoing PELD.

## Background

As a standard full-endoscopic surgical strategy for the treatment of lumbar disc herniation, percutaneous endoscopic lumbar discectomy (PELD) has become an alternative minimally invasive procedure to open lumbar microdiscectomy [1–3]. In PELD, patients are placed in the prone position, and the loose herniated nucleus pulposus is clipped out under endoscopic guidance with continuous irrigation. Local anesthetic infiltration (LAI) is recommended for PELD by most surgeon because surgeon can detect nerve root injuries in time [4]; however, under LAI, some patients cannot tolerate the pain caused by the instrument insertion, resulting in operative anxiety, pain-induced stress and inflammatory reactions. Then, opioids are added to prevent these conditions from occurring; this creates a challenge for anesthesiologists to assess the airway of patients in the prone position, so the implications of LAI in PELD remain contentious [4].

Dexmedetomidine (DEX), a new-generation, highly selective α_2_ adrenergic receptor agonist, is widely used in spine surgery due to its efficacy to achieve equilibrium between an effective analgesia and sedation regimen, and its minimal effect on the respiratory system [5–7]. However, the efficacy of DEX administration combined with LAI during PELD has not been described.

Oxidative stress and inflammatory reactions are two major risk factors among the serious complications impacting patient outcomes and recovery [8]. It is known that the level of inflammatory factors in the internal environment can directly reflect the stress state of the organism and an increase in the level of pro inflammatory cytokines, including tumor necrosis factor-α (TNF-α) and interleukin-6 (IL-6), is an early feature of acute injury. Glutathione peroxidase (GSH-PX) is an intracellular antioxidant enzyme that reduces hydrogen peroxide to limit its harmful effects. It specifically catalyzes the reduction of hydrogen peroxide with reduced glutathione (GSH), which can have a role in protecting the structural and functional integrity of cell membranes, and it is an important factor involved in the repair of damaged DNA during DNA synthesis [9]. Malondialdehyde (MDA) is a product of lipid peroxidation and the amount generated is proportional to the degree of peroxidation, which can reflect the degree of cell damage, which is one of the most recognized markers of oxidative stress. We studied inflammatory biomarkers IL-6 and TNF-α, and oxidative stress enzymes GSH-PX and MDA to reflect inflammatory and oxidative stress.

Perioperative management, such as alleviating oxidative stress and inflammatory reactions, can contribute to lowering the occurrence of organ dysfunction [8]. To better implement the concept of enhanced recovery after surgery (ERAS), it is particularly important to optimize the anesthesia method. Therefore, we investigated if DEX was superior in terms of maintenance of respiration, avoidance of oxidative stress, inflammatory reaction and analgesic.

## Materials and methods

### Study design, settings, and patients

This prospective, randomized trial was approved by the Medical Ethics Committee of the First Affiliated Hospital of Gannan Medical University (LLSC-2020121002), and all patients provided informed consent for all treatments and trials. All procedures performed in study involving human participants were in accordance with the ethics standards of the institutional and national research committee and with the 1964 Helsinki Declaration and its later amendments or comparable ethics standards. The study was conducted and reported in accordance with the Consolidating Standards of Reporting Trials (CONSORT) 2010 statement. This study was registered at www.chictr.org.cn. (ClinicalTrials.gov: ChiCTR2100044715; registered Study Chair: Reai Shan; registration date: 26/03/2021).

This study included adult patients with American Society of Anesthesiologists physical status (ASA) I/II who were scheduled to undergo elective PELD from June 2018 to March 2021. Patients with contraindications to LAI, allergies to any of the drugs planned to be administered, infection at the puncture site, or who had severe hypertension or diabetes, previous spine surgery, severe spinal stenosis, deranged liver function, cardiovascular impairments including preexisting heart block or compromised left ventricular function (defined as an ejection fraction < 45%) were excluded from the study. Surgeons and anesthesiologists were aware of the study being conducted but were blind to participant’s allocation.

### Study protocol

Patients were assigned randomly to receive either intravenous DEX sedation throughout the surgery (Group A) or the same volume of normal saline intravenously (Group B) using a computer-generated table of random numbers. The patient’s group allocation was concealed using a sequentially numbered, sealed opaque envelope, which was opened only by a separate investigator who prepared the infusion solution before the surgery. The day before surgery, patients were taught assessment of their pain using a visual analog scale (VAS), which ranged from 0 (no pain) to 10 (worst possible pain). The operations were performed by a single qualified surgeon (blinded to the study protocol) using the same technique. Intravenous access was secured before the patient’s arrival in the operating room.

In the operating room, standard monitoring was applied. All patients were provided with nasal catheter oxygen inhalation intraoperatively. DEX was diluted with normal saline to obtain a concentration of 4 μg·mL^− 1^, patients received 1 μg·kg^− 1^ of intravenous DEX for 10 min as a loading dose, followed by continuous infusion at a rate of 0.5 μg·kg^− 1^·h^− 1^ throughout the surgery (Group A), and patients in Group B received the same volume of normal saline. After the infusion of the loading dose, all patients received LAI in the surgical site using 10 ml of 1% lidocaine (Suicheng Industrial, China) and 10 ml of 0.75% ropivacaine (Qilu Industrial, China) by the same surgeon. Intravenous DEX or normal saline administration was stopped at the start of skin closure. During this procedure, if the patients felt significant pain upon incision (VAS > 3), 5–10 ml of 1% lidocaine was infiltrated at operation site as rescue analgesic. Venous blood samples were taken at T_0_ (baseline), T_5_ (at the end of surgery) and T_6_ (24 hours after surgery) to detect serum levels of IL-6, TNF-α by enzyme-linked immunosorbent assay (ELISA), and MDA and GSH-PX by colorimetric methods.

**Fig. 1:**
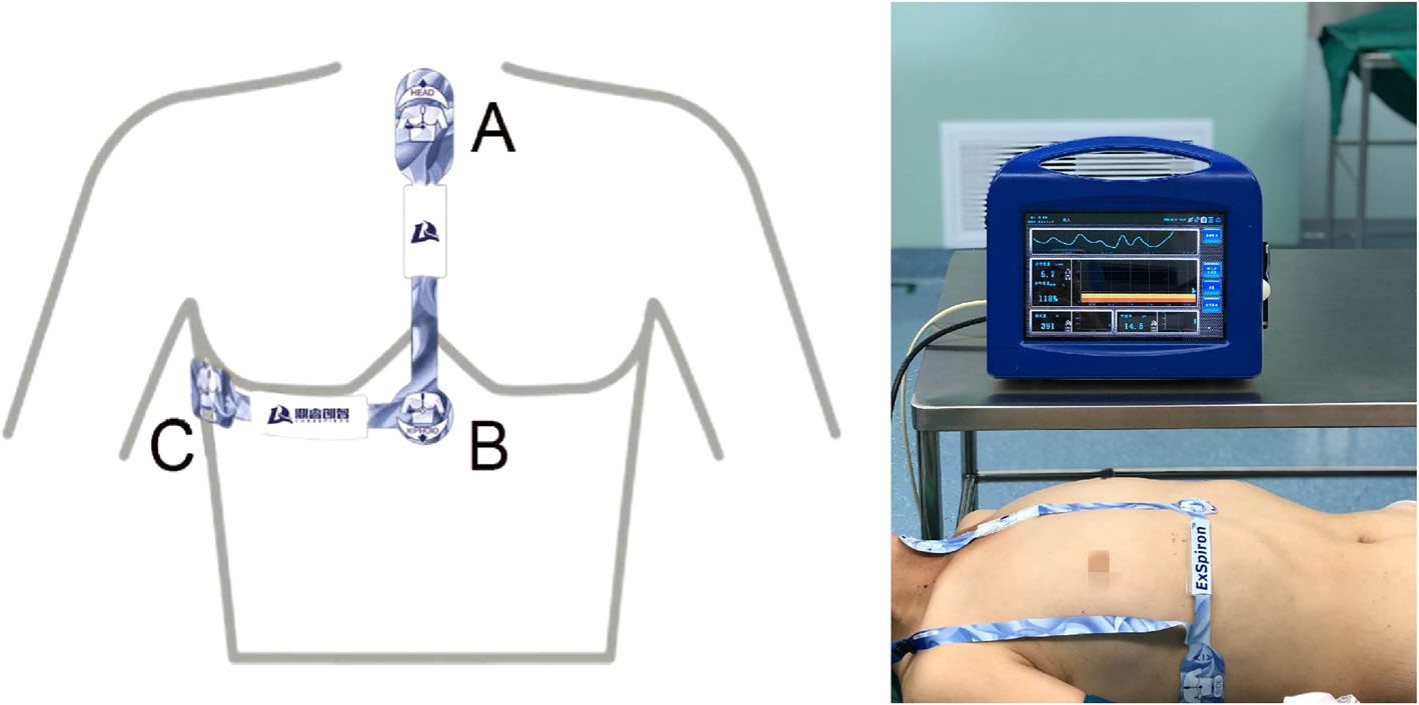
Standard PadSet Placement. A non-invasive Respiratory Volume Monitor (RVM, ExSpiron, Respiratory Motion, Inc.) that provides continuous, real-time, non-invasive measurements of MV, TV and RR. Figure shows standard electrode placement. One electrode is placed at the sternal notch, another is placed on the xiphoid and the third is placed in the right mid-axillary line at the level of the xiphoid

A Food and Drug Administration-approved, noninvasive, bioimpedance-based respiratory volume monitor (RVM; ExSpiron, Respiratory Motion, Inc., Waltham, MA) was used to provide real-time continuously respiratory data, including minute ventilation (MV), tidal volume (TV), and respiratory rate (RR). The predicted MV (MV_PRED_) for nonintubated patients, representing the expected MV during quiet respiration in the awake period, was calculated based on body surface area (BSA) and patient sex. The RVM collected bioimpedance traces via an electrode padset placed in the recommended positions: at the sternal notch (A), xiphoid (B), and right midaxillary line(C) at the level of the xiphoid (Fig. 1). The electrode padset was applied in a fashion similar to that of standard electrocardiogram electrodes.

Intraoperative hypotension (defined as a > 20% decrease in systolic pressure from baseline) was treated with 5–10 mg of intravenous ephedrine; bradycardia (defined as a heart rate < 45 beats min^− 1^) was treated with 0.5 mg of intravenous atropine. In case of MV less than 40%MVpred sustained for 1 min or longer plan was to manage the patient with tracheal intubation after changing position to supine.

### Measurement values

Data were recorded as the primary outcomes, including the SpO_2_, MV, VT, and RR at the following time points: T_0_, baseline; T_1_, at the end of bolus infusion of DEX/saline; T_2_, before the skin incision; T_3_, at the skin incision; T_4_, 30 min after skin incision; T_5_, at the end of surgery; and T_6_, 24 hours after surgery. The patients were asked to assess their pain at various time points of the surgical procedure from T_0_ to T_6._

### Statistical analyses

Sample size estimates were based on previous experience; serum concentrations of TNF-α at 24 hours after surgery was 75.74 ng/L with a standard deviation of 10.39. Assuming a two-sided test with an a error of 0.05, a b error of 0.2, and a dropout rate of 10%, 16 patients were required in each group to detect a difference of ≥20% in the mean value between the two groups [10].

Statistical analysis was performed using GraphPad Prism 8 (GraphPad Software, San Diego, CA, USA). Data are expressed as the mean ± standard deviation or the median (interquartile range). Data were analyzed using repeated-measures analysis of variance (ANOVA), and intergroup differences at the same time point were analyzed using a two-sample *t* test. *P* < 0.05 was considered to indicate a statistically significant difference.

## Results

**Fig. 2:**
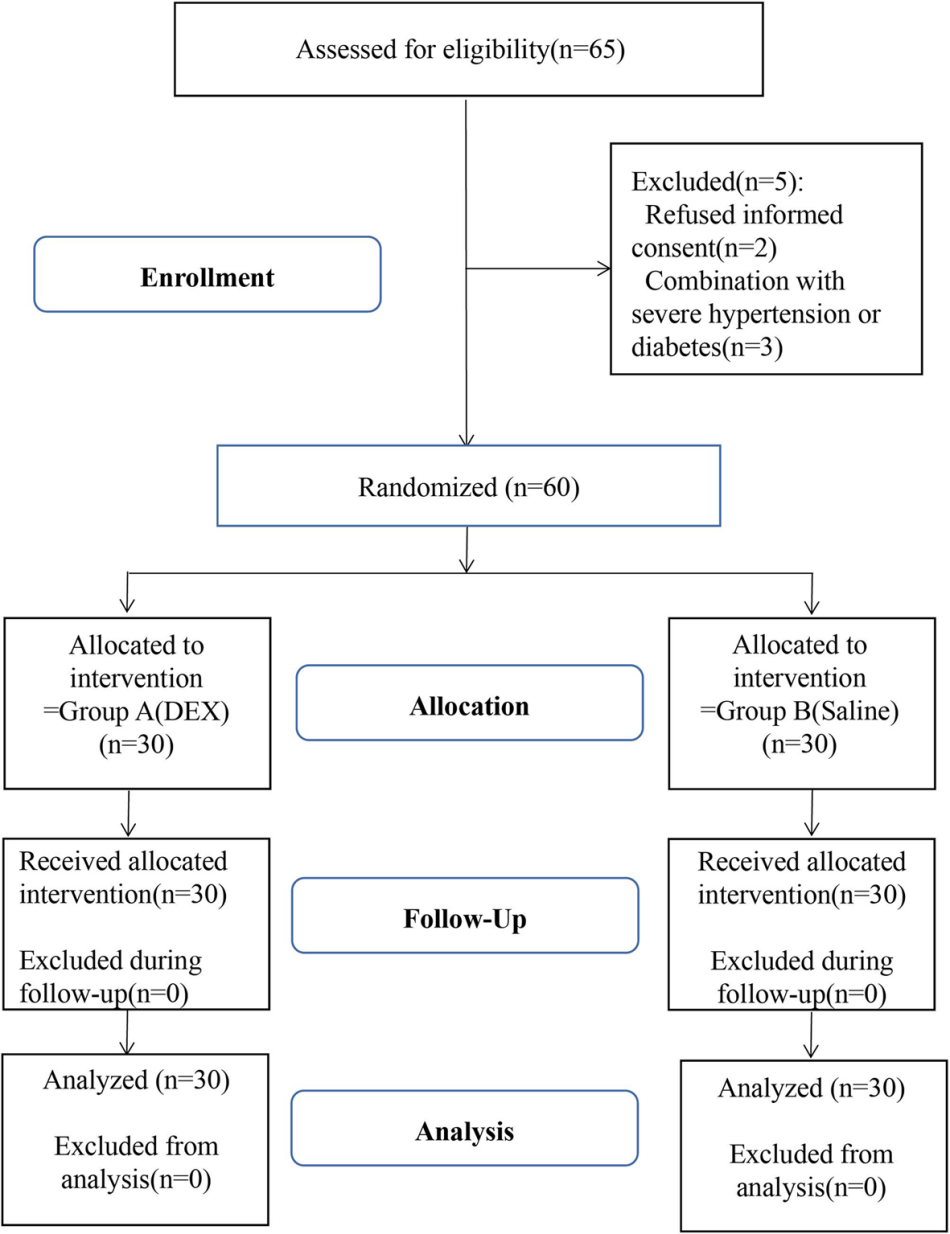
CONSORT flow diagram for the study

Initially, a total of 65 patients were enrolled, and 5 patients were excluded after applying the exclusion criteria: 2 patients refused to provide informed consent, and 3 patients had severe hypertension or diabetes. A total of 60 subjects were divided by randomization to complete the study and were analyzed (Fig. 2). There were no significant differences between the two groups in baseline characteristics (Table 1).

**Table 1:** Demographic data and surgical data

	Group A (*n* = 30)	Group B (*n* = 30)	*P* value
Gender, M/F	24/6	20/10	0.243
Age, years	71.43 ± 5.55	69.73 ± 6.07	0.262
Weight, kg	57.34 ± 11.15	58.25 ± 9.49	0.7329
ASA,(I/II)	5/25	3/27	0.4475
Intraoperative infusion, ml	1126.67 ± 383.21	1150.00 ± 289.17	0.791
Operation time, min	67.17 ± 12.79	62.53 ± 13.48	0.177

Data are expressed as mean ± standard deviation; Group A: DEX group; Group B: Saline group

### Respiratory variations

**Fig. 3:**
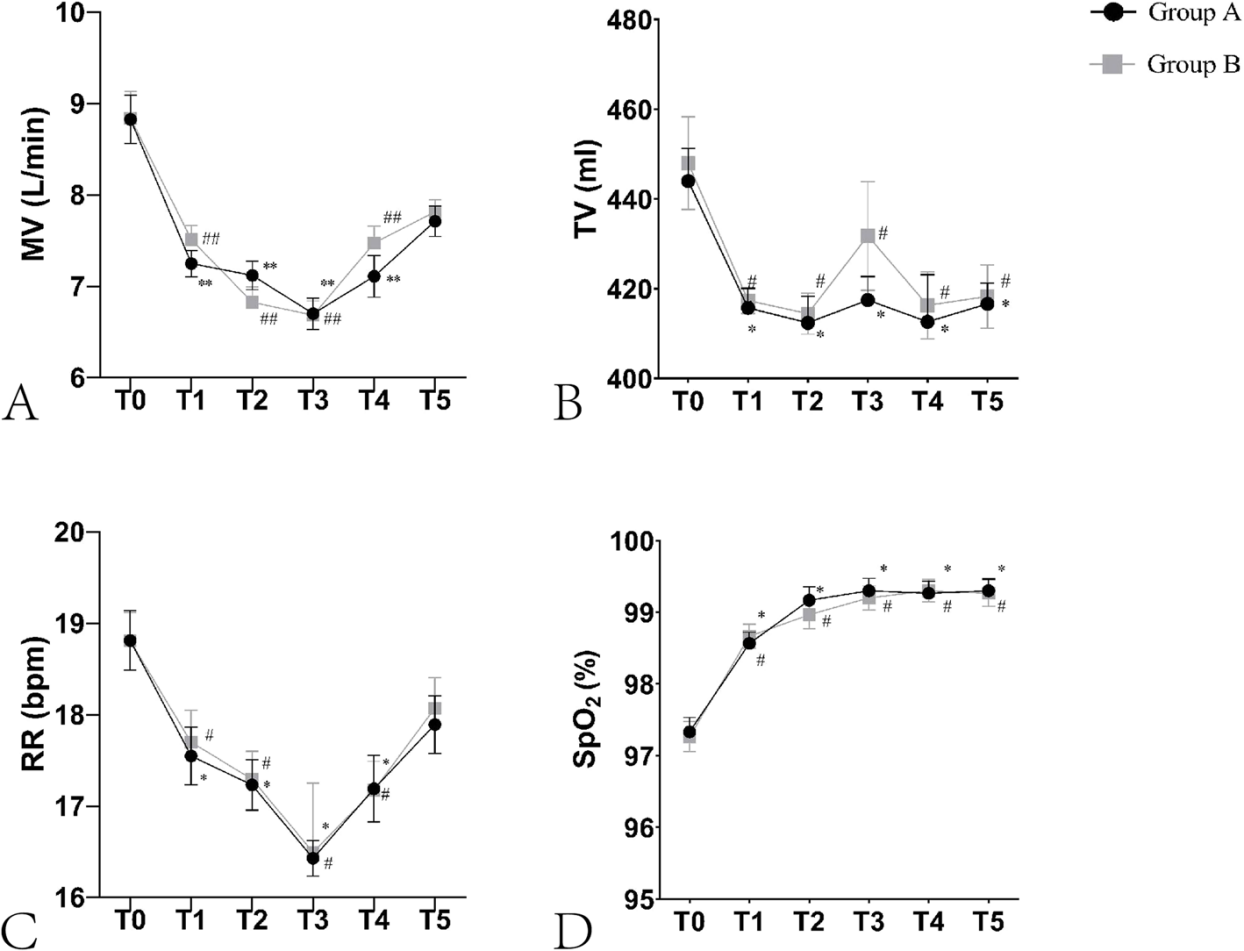
Changes of Respiratory Variations (mean ± SEM). **a** Trends in MV. **b** Trends in TV. **c** Trends in RR. **d** Trends in SpO2. There is no significant difference between groups but only between different timepoints within one group. Group A: DEX group; Group B: saline group; T0, baseline; T1, at the end of bolus infusion of DEX/saline; T2, before the skin incision; T3, at the skin incision; T4, 30 min after skin incision; T5, at the end of surgery. In Group A, vs T0 of Group A, ^*^P < 0.05, ^**^P < 0.01; In Group B, vs T0 of Group B, ^#^*P* < 0.05, ^##^*P* < 0.01

To assess the effects of procedural sedation using intravenous DEX on the patients’ respiratory drive when patients were placed in the prone position, the respiratory parameters (MV, TV, RR and SpO_2_) measured during PELD were analyzed. Figure 3 summarizes the recorded trends in MV, TV, RR and SpO_2_ and the variables for MV, TV, RR and SpO_2_ showed no significant differences between the two groups at any time point (*P* > 0.9999). However, in Group A, there were statistical differences from T_1_ to T_5_ compared that to T_0_ (*P <* 0.001). It’s the same with group B (*P <* 0.001).

Interestingly, the decrease in MV, TV, and RR was accompanied by a significant increase in SpO_2_ from 97.33 ± 1.09% to 99.30 ± 0.95% in Group A (*p* < 0.05) and 97.27 ± 1.14% to 99.20 ± 0.92% in Group B (*p* < 0.05).

### Comparison of VAS

There was no significant difference in the VAS at T_0_ and T_6_ between the 2 groups (*p* > 0.05); however, compared with Group B, there were significantly lower VAS from T_2_-T_5_ in Group A (*P* < 0.0001) (Table 2). Overall VAS scores for lumbar disc herniation induced pain showed a significant improvement in both the groups from 24 hours after the surgery (*P* < 0.001).

**Table 2:** Comparision of VAS

	Group A (*n* = 30)	Group B (*n* = 30)	*P* value
T_0_	6.70 ± 1.37	6.90 ± 1.27	0.745
T_2_	2.57 ± 0.86	3.93 ± 0.83^**^	<0.01
T_3_	2.80 ± 0.85	4.50 ± 0.63^**^	<0.01
T_4_	2.43 ± 0.56	5.73 ± 0.87^**^	<0.01
T_5_	3.00 ± 0.83	5.03 ± 0.81^**^	<0.01
T_6_	1.70 ± 0.84^@@^	2.13 ± 0.97^##^	0.262

Data are expressed as mean ± standard deviation;T_0_, baseline; T_2_, before the skin incision; T_3_, at the skin incision; T_4_, 30 min after skin incision; T_5_, at the end of surgery; T_6_, 24 hours after surgery. Group A: DEX group; Group B: Saline group;

**P < 0.01 vs Group A; In Group A, ^@@^*P* < 0.01 vs Group A at T_0_; In Group B, ^##^*P* < 0.01 vs Group B at T_0_

### Serum concentrations of GSH-PX, MDA, IL-6 and TNF-α

**Fig. 4:**
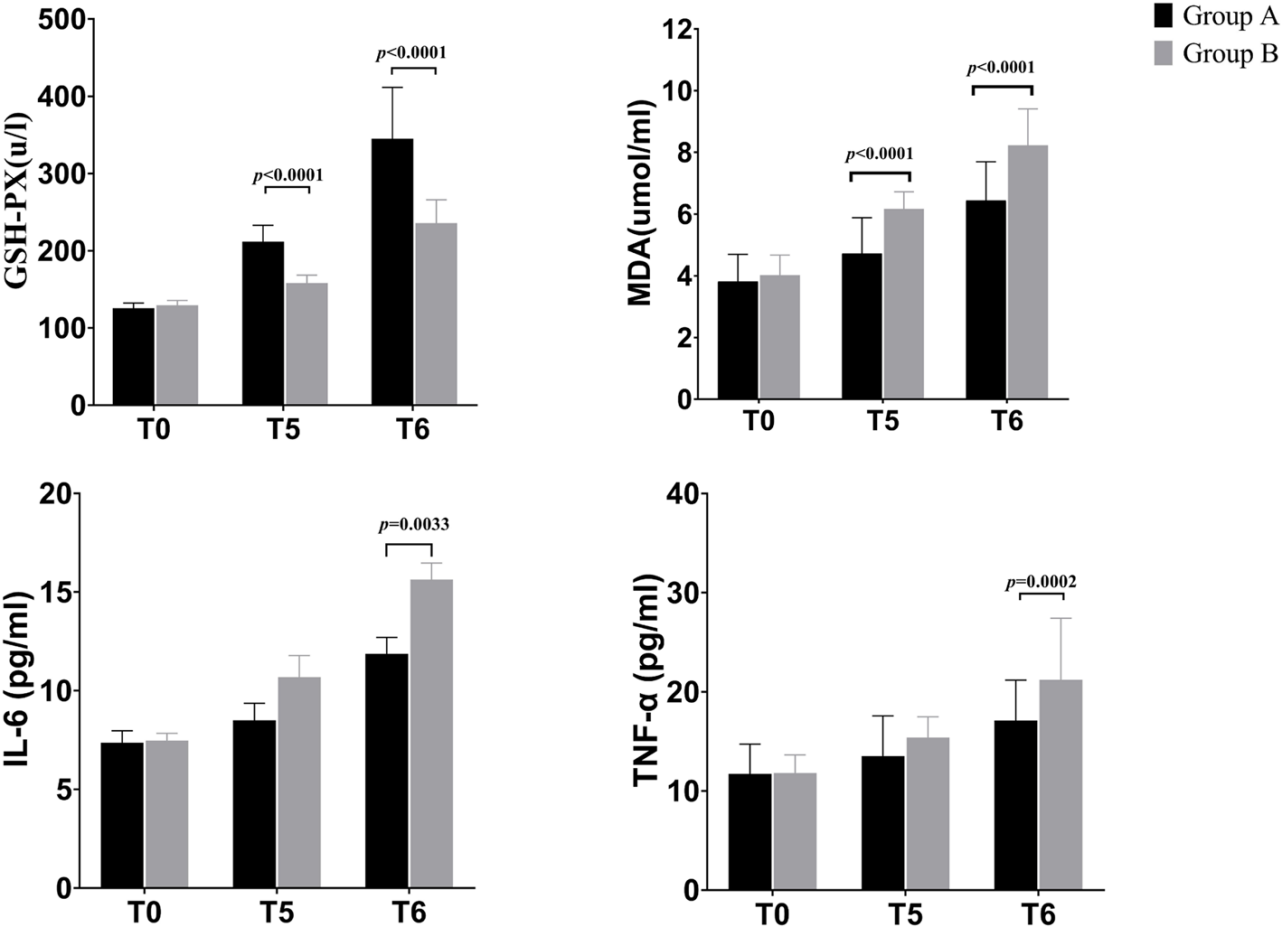
Serum concentration of GSH-PX, MDA, IL-6 and TNF-α (mean ± SD) were investigated in each sample for the two groups (Group A and Group B). **A** DEX group; **B**: saline group; T0, baseline; T5, at the end of surgery; and T6, 24 hours after surgery

There was no significant difference in T_0_ (the baseline values of inflammatory biomarkers and oxidative stress biomarkers) between the two groups (*P* > 0.05). Compared with T_0_, the levels of GSH-PX, MDA, IL-6 and TNF-α in both groups increased at T_5_ and T_6_ (*P* < 0.05). Compared with Group B, the levels of GSH-PX in Group A were significantly higher at T_5_ and T_6_ (*P* < 0.001) and serum levels of MDA were lower at T_5_ and T_6_ (*P* < 0.001). In addition, Group A exhibited lower serum levels of IL-6 (*P* = 0.0033) and TNF-α (*P* = 0.0002) at T_6_ than Group B (Fig. 4).

### Adverse reactions

No patients required endotracheal intubation for respiratory support, and there was no cases of MV less than 40% of the MV_PRED_ that were sustained for a period of 1 minute or longer. No patients required atropine for bradycardia or ephedrine for hypotension. No patients suffered cerebrospinal fluid leakage, nerve root injury, intraspinal hematoma, postoperative neuritis or other related complications.

## Discussion

This study demonstrated that DEX administration in non-intubated, spontaneously breathing patients undergoing PELD was safe and effective, although the patients were prone-positioned. Moreover, intravenous DEX could alleviate oxidative stress response and inflammatory reactions, which is of great significance for the concept of ERAS.

A novel RVM has been developed to continuously measure and display the MV, TV, and RR to monitor ventilation clinically, and a previous study demonstrated the accuracy of the RVM when compared with ventilators, as well as the accuracy of the RVM when compared to spirometry measurements [11]. Iwata and colleagues [12] demonstrated that DEX is excellent at preserving airway patency and preventing respiratory compromise, but the result of their study was based on SpO_2_ instead of MV, emphasizing the need for continuous ventilation monitoring in non intubated patients cannot be overemphasized. In previous studies respiratory parameters were quantified during the administration of midazolam and propofol and the postoperative administration of opioids, but there was a lack of data during the administration of intravenous DEX [13–15]. In our study, we found that compared with T_0_, the MV, TV, and RR values of the two groups declined from T_1_ to T_4_, and we hypothesize this may be related to the prone position resulting in chest and abdominal organ compression, impaired diaphragmatic activity and thereby, reduced ventilation. Although ventilatory parameters decreased, but their decline was insufficient to reduce SpO_2_ and we propose such reduction in ventilatory parameters to some extent, does not decrease oxygen saturation.

We defined respiratory depression as an MV less than 40% of the MV_PRED_ sustained for 1 minute or more. This criterion was originally based on the Acute Respiratory Distress Syndrome Network protocol for weaning patients off mechanical ventilation, which suggests that adequate ventilation associated with successful extubation is greater than 40% of the predicted value for normal respiratory volumes, and it was subsequently used to define inadequate ventilation to risk-stratify patients in the post anesthesia care unit (PACU) [16]. Previous studies have demonstrated that real-time MV measurements can be used to identify the patients at risk for opioid-induced respiratory depression and obstructive breathing patterns in the PACU, where patients with an MV less than 80% of their predicted MV prior to opioid administration had reductions to a critical level of less than 40% of the predicted MV after opioid administration [14, 17]. Similarly, we propose that it is feasible to identify the risk, and there were no patients with an MV less than 40% of their MV_PRED_ that was sustained for a period of 1 minute or longer in this study, which reveals that patients are less likely to develop respiratory depression in the postoperative period when given DEX during surgery.

To the best of our knowledge, this is the first ventilation monitoring study using an RVM to assess the respiratory status of patients receiving intravenous DEX. The advantage of using an RVM is that respiratory data can be monitored stably and reliably without patient discomfort, regardless of their body movements or position.

DEX has been popularly used for procedural sedation, however, its benefits and risks during PELD remain undetermined. Epidural anesthesia (a low concentration of ropivacaine) and LAI, instead of general anesthesia, were recommended by most surgeons for PELD because the surgeon can obtain feedback from the patient to avoid nerve injury [18]. Our study demonstrated that no significant difference was found in complications including cerebrospinal fluid leakage, nerve root injury, intraspinal hematoma and postoperative neuritis between the two groups, which confirms the safety of DEX. DEX exerts its hypnotic action through the selective activation of central pre- and postsynaptic alpha-2 adrenergic receptors in the locus coeruleus. Akeju et al. assumed that the altered arousal states induced with the administration of DEX neurophysiologically approximate natural sleep, and he termed this “biomimetic sleep” [19]. The pharmacological effect that produces biomimetic sleep makes it suitable for surgeons to obtain feedback from the patient to avoid nerve injury. As the result shown above, the administation of DEX did not produce respiratory depression and various other complications as stated above.

In addition, stress, such as from surgical procedures and intraoperative pain, can cause the release of a large number of biomarkers, such as TNF-α and IL-6 as well as oxidative stress enzymes GSH-PX and MDA, further activating neutrophils and monocytes, which can indirectly lead to organ dysfunction [8]. Recent studies found that DEX has an anti-inflammatory effect by reducing the levels of inflammatory cytokines. Increasing evidence suggests a critical role of TNF-α and IL-6 in the pathogenesis of pain including disc herniation-associated radicular pain and acute and persistent inflammatory pain [20]. Besides, studies have shown that oxidative stress is responsible for onset or progress of many diseases. A small but growing number of clinical studies have shown a positive correlation between high oxidative stress in the perioperative period and postoperative complications in patients undergoing major surgery, including liver, lung resections, and cardiac surgery [21–23]. Thus, the recovery can be evaluated by the changes in oxidative stress indicators. This study shows that DEX can markedly attenuated the increases in IL-6, TNF-α and MDA, meanwhile, it signifcantly increase the production of GSH-PX.

The significant reduction in the VAS in Group A reveals that DEX could provide analgesia during PELD so as to attenuate inflammatory response and pain-induced oxidative stress. According to the previous study, perioperative DEX administration can effectively reduce the generation of inflammatory mediators in the plasma, which relys on TLR4/MyD88/MAPK/ NF-κB signaling pathway and PI3K-Akt-GSK-3βsignaling pathway [24, 25]. In addition, through amelioration of HMGB1-NLRP3 inflammasome-AMPK signaling could DEX play protective action [26] and DEX has STAT3-dependent anti-inflammatory effects to attenuate the tissue inflammatory cascade response by modulating the JAK2/STAT3 signaling pathway [27]. Moreover, DEX administration attenuated the systemic inflammatory response through the cholinergic anti-inflammatory pathway involving vagus- and α7nAChR-dependent mechanisms [28].

This study has several limitations. First, we did not determine the optimal dose of dexmedetomidine to reach the balance between attenuation of various biomarkers and sedation, so more studies are needed to recommend the optimal dose of dexmedetomidine. Another limitation to the study is that the amount of perioperative local anesthetics was not analysed. Third, it is a singlecentre randomized trial, the results of this study need to be further confirmed by multi-centre studies.

## Conclusion

DEX administration does not produce respiratory depression or any other serious side effects in comparison to placebo (saline) in patients undergoing PELD under LAI. It also provides analgesia so as to alleviate oxidative stress and inflammatory reactions. Therefore, we recommend the use DEX in patients undergoing PELD.

## Data Availability

The datasets generated and analysed during the current study are not publicly available due do not we have consent from all patients to publish this data but the datasets used and analyzed during the current study are available from the corresponding author on reasonable request.
